# 10th Anniversary of *Biomedicines*—Advances in Endothelial Dysfunction

**DOI:** 10.3390/biomedicines13061403

**Published:** 2025-06-07

**Authors:** Francesca Schinzari, Carmine Cardillo, Manfredi Tesauro

**Affiliations:** 1 Department of Aging, Fondazione Policlinico Universitario Agostino Gemelli IRCCS, 00168 Roma, Italy; francesca.schinzari@policlinicogemelli.it; 2 Department of Translational Medicine and Surgery, Università Cattolica del Sacro Cuore, Largo A. Gemelli 8, 00168 Roma, Italy; 3 Department of Systems Medicine, Università Tor Vergata, 00133 Roma, Italy; mtesauro@tiscali.it

The vascular endothelium serves as an essential interface between tissues and the bloodstream, playing a key role in the regulation of blood flow. In its normal state, the endothelium inhibits blood coagulation, vasoconstriction, and inflammation, while upholding selective barrier functions. This carefully controlled set of characteristics can quickly adapt to trigger a range of responses necessary to stop hemorrhage from injuries or activate innate and adaptive immune mechanisms for tissue repair and defense against pathogens [1].

In its resting state, the endothelial cell employs various strategies to prevent thrombosis and encourage fibrinolysis, including the presence of anticoagulant heparan sulfate proteoglycans. Nitric oxide, the main endothelium-dependent vasodilator, and prostacyclin produced by the endothelial cells help to inhibit platelet aggregation and promote vasodilation. The protein thrombomodulin, found on the cell surface, binds to thrombin, imparting anticoagulant properties to this typically pro-coagulation factor by allowing it to activate protein C, which subsequently inactivates coagulation factors through a proteolytic process. Moreover, the resting endothelium enhances fibrinolysis by displaying plasminogen activators on its surface [2] (Figure 1, top panel).

Endothelial function can swiftly transition from a homeostatic condition to a potentially harmful defensive state. In response to pro-atherosclerotic risk factors, such as diabetes, dyslipidemia, or hypertension, or to pro-inflammatory cytokines released during viral infections or sepsis, the endothelium may produce tissue factor, a strong pro-coagulant, as well as plasminogen activator inhibitor-1 (PAI-1), which inhibits fibrinolysis. Many endothelial cells also secrete von Willebrand factor, a crucial component in thrombus formation. Pro-inflammatory cytokines prompt endothelial cells to boost their cytokine production and to express adhesion molecules that bind leukocytes and release chemoattractant chemokines, which help guide these inflammatory cells to cross the endothelium. In contrast to the low levels of leukocyte movement during a homeostatic state, inflamed endothelial cells display adhesion molecules that attract granulocytes, and the localized release of chemoattractants guides the migration of inflammatory cells into tissues. Furthermore, inflammation can induce vasoconstriction, as superoxide anions generated by activated leukocytes can interact with nitric oxide to create the highly pro-oxidative compound peroxynitrite, which can further exacerbate tissue damage (Figure 1, bottom panel). Also, the potent vasoconstrictor endothelin-1 produced by endothelial cells can enhance inflammation [1,2].

The Special Issue “10th Anniversary of *Biomedicines*—Advances in Endothelial Dysfunction” includes a collection of manuscripts from renowned investigators in the field, providing a broad survey of the impact of the endothelium on several physiological functions and pathophysiological states. Of particular interest is the work presented by Kreslova et al. regarding the circulating biomarkers of endothelial dysfunction in children with the multisystem inflammatory syndrome associated with COVID-19 [3], and the review by Sekulovski et al. about the relationship between endothelial dysfunction and thrombophilia in pregnant women with COVID-19 [4]. In infection with SARS-CoV-2, it has been demonstrated that altered endothelial function not only participates in the multiorgan adverse effect during the acute phase of COVID-19, but might also contribute to some long-term consequences post-COVID-19, such as the postural orthostatic tachycardia and “brain fog” syndromes [5]. Specifically, the systemic release of inflammatory cytokines during acute COVID-19 promotes thrombus formation, increased trans-endothelial leukocyte trafficking and migration of inflammatory cells into tissues, and the activation of a particular type of cell death in granulocytes leading to the formation of extracellular neutrophil traps (NETs), which further promote thrombosis. COVID-19 is not the only viral infection associated with marked endothelial activation secondary to enhanced cytokine secretion. Thus, such a mechanism, together with monocyte activation and persistent immune dysregulation, has also been evoked to explain the development of cardiovascular complications in individuals with HIV infection [6]. In people living with HIV, the detrimental endothelial effect of the virus itself may compound those of combination antiretroviral therapy (ART). Thus, on the one hand, ART improves the life expectancy of these patients, but, on the other hand, it increases the risk of cardiovascular events, mainly due to protease inhibitory mechanisms. Prolonged ART may also result in dysregulated endothelial mitochondrial balance and genomic instability, leading to premature vascular aging [7].

Various factors, including oxidative stress, telomere erosion, DNA instability and metabolic imbalance can trigger endothelial cell senescence. The activation of specific senescence-associated secretory phenotypes, resulting in a chronic inflammatory microenvironment, and the up-regulation of the mammalian target of rapamycin (mTOR) pathway, which promotes a proliferative arrest, have been identified as pivotal mechanisms in cardiovascular aging [8]. As the detrimental secretome of senescent cells stimulates survival pathways that can harm neighboring cells, activate pro-survival pathways, and reduce regenerative capacity, the accumulation of senescent cells in the vasculature impairs the regeneration of endothelial cells, which already have limited proliferative potential [9]. The recent identification of molecular pathways and biochemical markers of vascular cell senescence has incited investigations into potential interventions to slow this process, thereby preserving cardiovascular health and extending lifespan. Ongoing studies are testing pharmacological approaches based on senolytics, substances that specifically eliminate senescent cells, or senomorphics, agents neutralizing the senostatic effects of the senescence-associated secretome. These therapies have shown promise in reducing the senescence-related endothelial impairment, but further evidence is needed to fill the knowledge gaps in their clinical application for endothelial rejuvenation and the revitalization of the cardiovascular system [9]. The prevention or reversion of vascular aging is one of the main benefits of physical activity and exercise in improving general health and well-being. As aerobic exercise has been shown to improve endothelium-dependent vasodilator function by reducing inflammation and oxidative stress, while concurrently improving mitochondrial metabolism, it is reasonable to believe that these mechanisms might underline the favorable endothelial effects of exercise-based interventions on vascular aging and cardiovascular protection more generally [10]. Because the effects of exercise may be influenced by multiple factors, more precise identification is needed of those exercise-regulated molecules that are biomarkers of endothelial health or are involved in the cross-talk between the vasculature and other tissues during exercise. Thus, a deeper understanding of the signaling pathways involved in exercise benefits could undoubtedly contribute to the development of appropriate exercise protocols as part of a holistic strategy for improving cardiovascular health.

In addition to their classical functions in regulating blood flow to individual organs and exerting cardiovascular protection, endothelial cells (ECs) exert a variety of “unconventional” functions in tissue regeneration, angiogenesis, cancer biology, and immune regulation [11]. For example, ECs have been shown to play pivotal roles in the tumor microenvironment, where they contribute to the formation of new blood vessels (angiogenesis) to supply nutrients and oxygen to the tumor cells, contributing to tumor growth and metastasis; additionally, ECs may help to create an immunosuppressive environment, protecting the tumor from the immune response. Consequently, anti-angiogenic strategies targeting ECs have become part of an integrated therapeutic approach to improve cancer treatment outcomes. In the context of both organ and bone marrow transplants, some of the most dreadful complications, such as the vein occlusive disease of the sinusoid occlusion syndrome, diffuse alveolar hemorrhage, and transplant-associated thrombotic microangiopathy, are mediated by EC abnormalities. Because the endothelium is the first interface between donor and recipient immune cells, targeting immune–EC interactions might prevent organ rejection or graft-versus-host disease. The endothelium also seems important in regenerative medicine, as hinted at in the current Special Issue of Biomedicines in a review article by Rudnicka-Drozak et al. on the role of endothelial progenitor cells (EPCs) in neurovascular disorders [12]. EPCs are a population of circulating cells that migrate to areas of endothelial or vascular injury to repair them. As endothelial dysfunction is a relevant component of neurovascular disorders, the possible role of EPCs in these conditions has been extensively investigated, leading to the notion that decreased levels of EPCs are associated with worse disease outcomes and that EPC functionalities decline with the severity of the disease. These observations have inspired the use of EPCs as potential therapeutic agents. So far, EPCs appear safe and promising based on the results of pre-clinical studies conducted on their use in the treatment of Alzheimer’s disease and ischemic stroke, but clinical trials needed to confirm the applicability of this approach in patient care are lacking. Numerous unresolved questions still need to be addressed before considering the therapeutic use of EPCs in clinical practice. For example, studies using single-cell RNA sequencing have highlighted the heterogeneity of ECs in different tissues, identifying specific molecular signatures and functional properties unique to ECs from various organs [11]. This aspect demonstrates the diversity of EC transcriptional profiles in different organ systems, suggesting the necessity to preliminarily ascertain the mechanisms underlying organ-specific EPC differentiation.

In conclusion, even though this Special Issue of Biomedicines provides a wide overview of endothelial pathophysiology, many gaps in knowledge still exist, especially in the translation of our expanding understanding of EC biology into effective therapeutic approaches. Hopefully, this awareness will inspire further basic and clinical research to improve our current understanding of the field and provide effective endothelium-targeting therapies to patients.

## Figures and Tables

**Figure 1:**
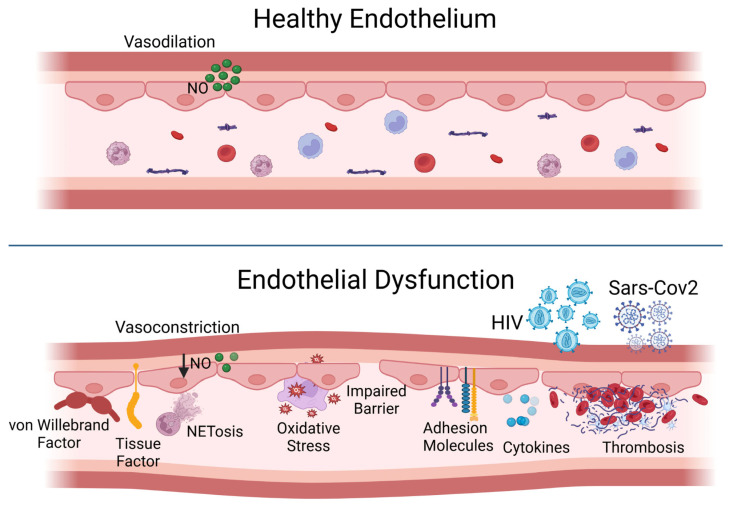
Under physiological conditions, the endothelium preserves vascular homeostasis through combined mechanisms, including vasodilation, anti-platelet and anti-thrombotic actions, fibrinolytic activity, and physical barrier, to prevent the extravasation of fluids and blood components. The damage of endothelial cells by cardiovascular risk factors, inflammatory cytokines, or pathogens can determine endothelial activation and dysfunction, resulting, in turn, in vasoconstriction, impaired fibrinolysis, pro-thrombotic milieu, and the loss of barrier functions. NO, nitric oxide; NET, neutrophil extracellular trap; HIV, human immunodeficiency virus; SARS-CoV-2, severe acute respiratory syndrome-coronavirus2.
